# Deciphering the molecular and cellular underpinnings of cognitive resilience in Alzheimer’s disease

**DOI:** 10.1002/alz.090374

**Published:** 2025-01-03

**Authors:** Isabel Castanho, Pourya Naderi Yeganeh, Carles A Boix, Hansruedi Mathys, Dmitry Prokopenko, Bartholomew White, Sarah Morgan, Christoph Lange, David A. Bennett, Lars Bertram, Rudolph E. Tanzi, Li‐Huei Tsai, Manolis Kellis, Winston Hide

**Affiliations:** ^1^ Beth Israel Deaconess Medical Center, Boston, MA USA; ^2^ Harvard Medical School, Boston, MA USA; ^3^ Massachusetts Institute of Technology, Cambridge, MA USA; ^4^ University of Pittsburgh, Pittsburgh, PA USA; ^5^ Massachusetts General Hospital, Charlestown, MA USA; ^6^ Queen Mary University of London, London, MA United Kingdom; ^7^ Harvard T.H. Chan School of Public Health, Boston, MA USA; ^8^ Rush University, Chicago, IL USA; ^9^ Center for Lifespan Changes in Brain and Cognition (LCBC), Department of Psychology, University of Oslo, Oslo Norway; ^10^ University of Lübeck, Lübeck Germany; ^11^ Picower Institute, MIT, Cambridge, MA USA

## Abstract

**Background:**

A significant proportion of individuals preserve cognitive function despite meeting neuropathological criteria for Alzheimer’s disease (AD) at autopsy, known as cognitive resilience. We aimed to define the molecular and cellular signatures of cognitive resilience against AD.

**Method:**

We integrated multi‐modal data from the Religious Order Study and Memory and Aging Project (ROSMAP), including bulk (n = 631) and multi‐regional single nucleus (n = 48) RNA sequencing. Subjects were categorized into AD, Resilient, and Control based on Aβ and tau pathologies, and cognitive status. We investigated genetic risk and transcriptomic changes in resilience and prioritized protected cell populations using genetic enrichment and cellular distribution estimation. We further characterized these populations using multiplex immunofluorescence.

**Result:**

Resilient individuals exhibited an intermediate genetic risk profile between AD and Control. Remarkably, only *GFAP* and *KLF4* were differentially expressed between Resilient and Control subjects in bulk tissue. *GFAP* was upregulated in Resilient astrocytes in the dorsolateral prefrontal cortex compared to AD and Control. Inhibitory and excitatory neurons displayed distinct brain region‐specific phenotypes in cognitive resilience. Somatostatin‐positive interneurons were enriched for genes linked to protective rare genetic variants and showed vulnerability in AD but not in Resilient individuals. Specific excitatory neuronal populations in the entorhinal cortex exhibited resilience‐like behavior and expressed genes previously associated with cognitive resilience.

**Conclusion:**

Our findings suggest that at the bulk tissue transcriptome level, cognitive resilience does not exhibit marked gene expression differences from healthy aging, despite significant Aβ and tau neuropathology. At the cellular level, specialized excitatory neuronal populations may drive cognitive resilience, while a specific subset of interneurons may play a crucial role in other forms of protection against AD‐associated neuronal vulnerability. This systematic study provides direct insight into the molecular basis of cognitive resilience, yielding targets offering the potential to convert natural protective systems into effective therapeutic interventions for AD.

